# Do the Colors of the Label and the Sweetening Agent Information Influence the Sensory Expectations Consumer? A Case Study with Skyr-Type Yogurt

**DOI:** 10.3390/foods11020167

**Published:** 2022-01-09

**Authors:** Cecília Teresa Muniz Pereira, Alessandra Cazelatto de Medeiros, Marcella Benetti Ventura, Dalva Muniz Pereira, Helena Maria André Bolini

**Affiliations:** 1 Department of Food Engineering and Technology, University of Campinas, Monteiro Lobato, 80, Campinas, São Paulo 13083-862, Brazil; ceciteresa@ifma.edu.br (C.T.M.P.); acmls@unicamp.br (A.C.d.M.); ella_mv@hotmail.com (M.B.V.); 2 Department of Educational Development, Federal Institute of Education, Science and Technology of Maranhão (IFMA), MA-340, KM 02, Povoado Lamengo, Caxias 65600-000, Brazil; dalva.pereira@ifma.edu.br

**Keywords:** consumer expectation, labeling, dairy products, natural sweeteners, concentrated yogurt

## Abstract

The objective of this research was to evaluate the sensory expectation and buying intention of consumers from different Brazilian regions for skyr-type yogurt based on the colors and sweetener on its label. Ten images of skyr mango yogurt labels were created varying in color (orange, white, yellow, blue, and green) and sweetening agent (sucrose and natural sweeteners). Consumers (151 consumers) were asked to rate their expectation for the ideal of sweetness, healthiness, acceptance, and buying intention. Labels containing the information “sweetened with sucrose” had a higher percentage of expectation of sweeter than ideal. Label color and sweetening agent had a significant effect on the expectation of acceptance, with a higher percentage for yogurt sweetened with natural sweeteners. There were not statistical differences (*p* > 0.05) between the labels regarding expected healthiness. Results also showed that consumers had a low level of familiarity with skyr-type yogurts, but it is presented as a healthy yogurt alternative.

## 1. Introduction

Sensory evaluation of food products involves several sensory modalities. The evaluation process starts before the actual tasting, through distal visual or acoustic perception and proceeds proximally with food intake [1].

Understanding the relative importance of attributes that influence food choice at the point of sale is important for the successful development of new products [2]. The sources that consumers have before consumption are usually classified as ‘extrinsic product’ cues, which are those that are somehow related to the food but are not physically a part of it such as labeling and packaging [3].

Packaging and labeling are important to capture the consumer’s attention and define their expectations related to the product [4]. Color, for example, can be found in almost all packaging and labels of food products, and the color information on these products serves both aesthetic and symbolic functions for consumers [5]. Food buyers can associate colors with sensory characteristics and healthiness, and depending on the color of the package/label it can trigger more or less favorable associations of flavor and health [6].

Excessive consumption of some foods and unhealthy food choices are at the root of one of the most urgent health concerns facing much of the developed world [7]. Immoderate sugar consumption is a public health problem associated with several non-communicable chronic diseases [8]. Thus, the use of natural sweeteners represents a strategy for reducing sugar while maintaining the health appeal of products such as skyr yogurt. Skyr is a yogurt of Icelandic origin which is subjected to centrifugation and ultrafiltration processes, resulting in a full-bodied and creamier texture [9]. This product is commonly associated with healthiness since it is a concentrated yogurt and, for this reason, it’s composition has higher contents of some nutrients (such as protein). The type of sweetening agent used in this food can impact its healthiness since, for yogurt consumers, one of the most important sensory attributes is flavor, including sweetness [10].

Some studies have shown how the association between extrinsic cues (such as color and package shape) and flavor can influence consumer expectations for different foods and beverages, such as wine [11], French fries [12], cold tea [13], and coffee [14]. In dairy products, how color and shape of the packaging influence consumer expectations of dairy desserts [15] has been studied, as well as consumer perceptions about three fermented dairy products through labeling [16] and the effects of familiarity on the expected taste of cheese [17]. In yogurts, the effects of the image used to communicate that a natural yogurt is sweetened on consumer expectations and willingness to buy [18] and the effects of the internal color of the packaging in post-buying and pre-consumption consumers behaviors [19] were studied.

Associations among color, shape, and flavor have been explored in a variety of food and beverage products [6,11,12,13,14,15,18,19]. The presence of information on the label regarding the type of yogurt, natural sweeteners, and coloring can make the consumer create sensory and health expectations during the choice even before tasting the product, making it important to analyze these factors. The information on the label directs the first purchase once the consumer’s attention has been drawn to the product. Thus, the sensory characteristics can meet expectations, making the product accepted in the market, or not capture consumer attention, leading to the consumer being less likely to buy the product. Therefore, the objective of this work was to verify the influence of label color and sweetener on skyr yogurt consumers’ expectations of flavor and healthiness as well as their perceptions and attitudes surrounding concentrated yogurts.

## 2. Materials and Methods

### 2.1. Stimuli

**Figure 1:**
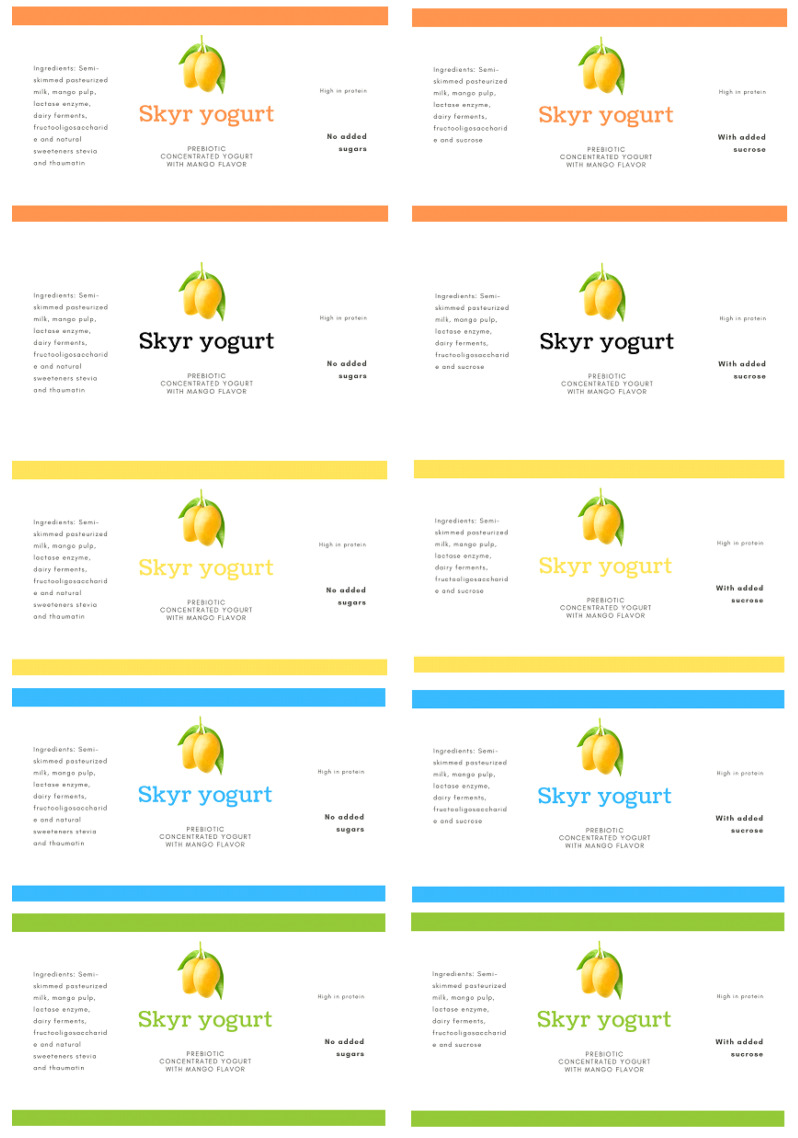
Representation of ten labels used in the study (L1 to L10).

The visual stimuli used in the experiment were representations of prebiotic skyr yogurt labels with mango pulp. The choice of presenting a flavored yogurt label was due to the fact that adding flavor to yogurt generally increases its acceptance [20], mainly popular tropical fruit such as mango. The design consisted of two types of sweetening agent (sucrose and natural sweeteners stevia/thaumatin) and five colors (orange, white, yellow, blue, and green), generating 10 label combinations. Figure 1 shows the elaborated labels used as stimuli for the research. The colors used on the labels were the most cited in a previous survey, where consumers were asked which colors would best represent a mango-flavored skyr-type concentrated yogurt label. Other label features, such as product name and image, were identical under all conditions. The 10 images were presented separately to the research participants (in a sequential monadic way) in balanced complete block design. All samples were coded with three-digit random numbers.

### 2.2. Participants

Participants were recruited through social networks and email lists, and 151 consumers from all regions of Brazil participated in the test. Participants signed an informed consent form before the evaluation. Most research participants were women (71.5%), aged 25–34 years (45.0%) (Table 1). The study was approved by the Research Ethics Committee of the State University of Campinas (UNICAMP), Brazil (CAAE: 91178118.0.0000.5404).

### 2.3. On-Line Research

The test was performed online using Compusense Cloud^®^ (Compusense Inc., Guelph, ON, Canada) with an average response time of 10 min. Research was divided into 3 distinct sessions. The first session contained socioeconomic and demographic questions (age, gender, marital status, resident status, education, and family income). The second session contained questions about yogurt consumption habits, consumption of concentrated yogurt, and preferences regarding yogurt flavor. In this session, participants also indicated the degree of familiarity with skyr-type and Greek-type concentrated yogurt. Answers were obtained through a 9-point structured scale, anchored to the left with “extremely unknown” and to the right with “extremely familiar” [21].

In the third session, participants were introduced to the labels using a balanced complete block design [22] in a sequential monadic way [23]. The attributes of yogurt for each label evaluated were: just-about-right sweetness, healthiness, acceptance, and willingness to buy. For sweetness, a 5-point ideal scale was used, anchored on the left with “Not sweet enough,” on the right with “too sweet” and “Just-about Right” in the center [24]. Healthiness and acceptance were assessed using a 9-point scale, with the healthiness scale anchored with “not healthy at all” on the left and “totally healthy” on the right; the acceptance scale was anchored with “extremely dislike” on the left and “extremely like” on the right. For buying intention, a 5-point scale labeled on the left with “certainly would not buy” and with “certainly would buy” on the right was used.

### 2.4. Data Analysis

Demographic and behavioral issues were analyzed in terms of frequency of choice. An analysis of variance (ANOVA) on average ratings of familiarity with concentrated yogurt was performed. The ideal sweetness and buying intention data were analyzed by their correspondent scores histogram. A repeated measures analysis of variance (ANOVA) was performed on the data to verify the effect of color and sweetener on consumer expectations regarding healthiness and acceptance. Sweetener (sucrose and stevia/thaumatin sweeteners) and color (orange, white, yellow, blue and green) of the packaging label design elements along with their interaction and participant effects were considered explanatory variables [25]. Honestly significant differences were calculated using Tukey’s test. Differences were considered significant when *p* < 0.05. Cluster analysis was performed using agglomerative hierarchical clustering based on Euclidean distances between consumers, and PREFMAP was used to construct preference map contour graphs. All data analyses were performed using XLSTAT software for Windows, version 2012.5 (Addinsoft, Paris, France).

## 3. Results and Discussion

### 3.1. Demographic and Consumption Data of the Study Participants

Most respondents were from the Northeast (43.7%) and Southeast (41.7%) regions, single (59.6%), with a postgraduate degree (73.5%), and a family income of 5 to 10 Brazilian minimum wages (31.1%) (Table 1). Consumption data revealed that there was a higher percentage of survey participants who reported consuming yogurt occasionally (37.1%), followed by consumers who reported consuming yogurt monthly (22.5%). Consumption was low compared to countries like Hungary [26], Switzerland [27], and United States [28]. Previous work confirms the low consumption of dairy products in Brazil, including yogurt, with significant variations depending on demographic and socioeconomic variables [28,29].

The main factor considered for most consumers when choosing a yogurt was flavor (76%), followed by price and brand. Consumers considered sensory characteristics the most important factor in food choice, and flavor was the main determinant of product acceptability and preference [30,31], which led consumers to conduct their choices using this parameter. In other dairy products, such as chocolate milk, flavor is also the main factor in the buying decision [32].

When asked about yogurt flavor preference, consumers indicated that fruit yogurts and sweetened fruit yogurts were preferred, confirming that adding fruit to yogurt increases its popularity and helps with marketing and the retention of consumer interest [20]. Fruit preparations, when added to yogurt, provide functionality, bringing with them the fruit’s natural sugars [33] which attract consumer attention and acceptance.

Regarding the level of familiarity of consumers with concentrated yogurt, skyr yogurt had a mean of 3.06 (equivalent to very unknown on the scale), differing significantly (*p* < 0.05) from Greek yogurt (mean of 6.82), demonstrating a low level of consumer familiarity with this type of yogurt. Skyr yogurt is a concentrated yogurt, characteristic of Iceland, having recently arrived on the Brazilian dairy market. The main characteristics of concentrated yogurts are their creamy texture and high protein content, which boosted sales of this type of yogurt, especially among consumers concerned about health and a diet rich in protein. This category in Brazil is mainly represented by Greek yogurt, which makes it better known, but interest in skyr yogurt has been increasing [34].

### 3.2. Expectation of Sweetness Ideal and Buying Intention

**Figure 2:**
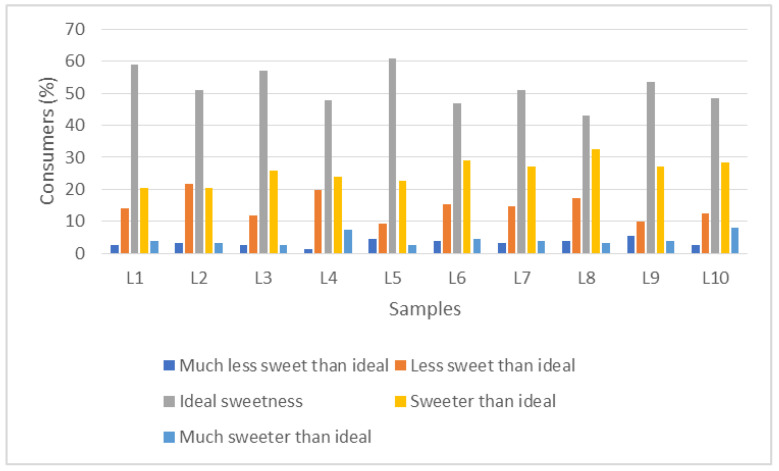
Histogram with the distribution of responses in the ideal test for the label samples. L1 (Orange color label with sweeteners), L2 (White color label with sweeteners), L3 (Yellow color label with sweeteners), L4 (Blue color label with sweeteners), L5 (Green color label with sweeteners), L6 (Orange color label with sucrose), L7 (White color label with sucrose), L8 (Yellow color label with sucrose), L9 (Blue color label with sucrose), L10 (Green color label with sucrose). Each column represents the mean percentage frequency ([the number of participants who selected each term from the scale] × 100/[the total number of participants]) for the ten labels.

Results for expected sweetness revealed that L5 (blue with stevia/thaumatin sweeteners) and L1 (orange with stevia/thaumatin sweeteners) labels reached the highest percentages of ideal sweetness (Figure 2). Labels sweetened with sucrose (L6, L7, L8, L9, and L10) had higher percentages of sweeter than ideal when compared to labels sweetened with stevia/thaumatin sweeteners. The expectation almost always existed before experiencing flavor, and when consumers form expectations, nutritional and ingredient information are taken into account through knowledge and associated memories [35]. Due to most of the sweetened yogurts available on the market generally having a high concentration of sucrose (around 12%), consumers may have associated this information with an expectation of above-ideal sweetness on yogurt labels with the information “with addition of sucrose”. Johansen et al. [36], in a study with reduced sugar and fat yogurts, found that consumer acceptance was generally increased with information about low/no sugar content of the products.

**Figure 3:**
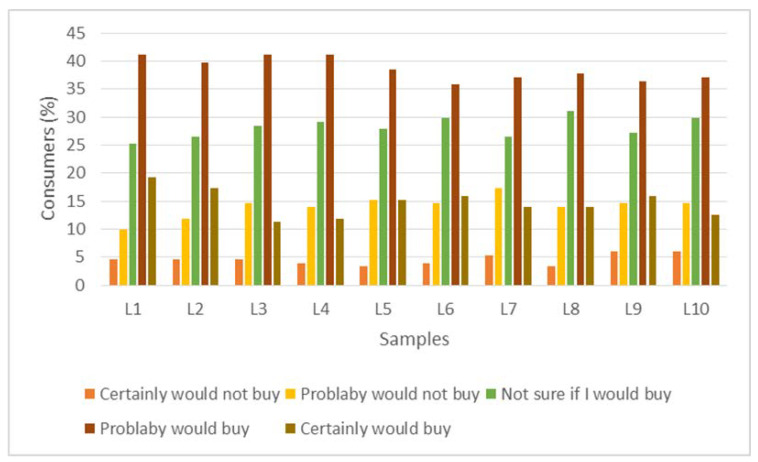
Histogram with the distribution of responses in the buying intention test for the label samples. L1 (Orange color label with sweeteners), L2 (White color label with sweeteners), L3 (Yellow color label with sweeteners), L4 (Blue color label with sweeteners), L5 (Green color label with sweeteners), L6 (Orange color label with sucrose), L7 (White color label with sucrose), L8 (Yellow color label with sucrose), L9 (Blue color label with sucrose), L10 (Green color label with sucrose). Each column represents the mean percentage frequency ([the number of participants who selected each term from the scale] × 100/[the total number of participants]) for the ten labels.

In general, labels had higher percentages for “probably would buy,” in relation to buying intention, for those sweetened with stevia/thaumatin sweeteners (Figure 3). Consumer buying intention was generally high when the claim of no added sugars and use of natural sweeteners is used. In protein drinks sweetened with natural sweeteners, the buying intention for drinks labeled as naturally sweetened was higher, not differing between consumer segments [37]. Oltman et al. [38] reported that the type of sweetener was the most important attribute for consumers of protein drinks, with naturally sweetened claims being more desirable. Li et al. [39] also found that chocolate drinks with natural sweeteners were among those preferred by parents when buying this product for their children.

### 3.3. Influence of the Sweetener and Label Color on the Expectation of Health and Consumer Acceptance

Label color and type of sweetener had a significant effect on acceptance (*p* < 0.05). The sweetener*color interaction was not significant for healthiness and acceptance.

**Figure 4:**
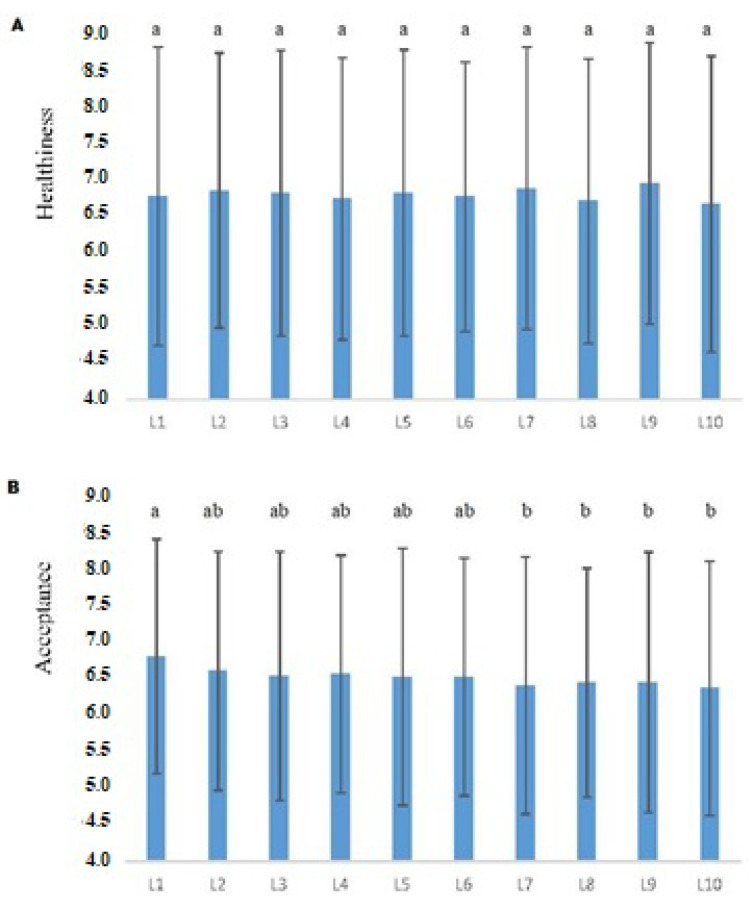
Mean as a Function of the color of the label and the type of sweetener. (**A**) Healthiness and (**B**) Acceptance. Error Bars Represent Standard Errors. Means with common letters indicate that there is not a significant difference between samples (*p* < 0.05) from Tukey’s Honestly Significant Difference procedure (HSD). Samples: L1 (Orange color label with sweeteners), L2 (White color label with sweeteners), L3 (Yellow color label with sweeteners), L4 (Blue color label with sweeteners), L5 (Green color label with sweeteners), L6 (Orange color label with sucrose), L7 (White color label with sucrose), L8 (Yellow color label with sucrose), L9 (Blue color label with sucrose), L10 (Green color label with sucrose) (X-axis). The scale from 0 to 9 was limited to 4 for better visualization (Y-axis).

In packaging design, color is seen as one of the most effective resources in the food industry, and both consumers and manufacturers often associate healthy and nutritious foods with packaging in green or blue colors, for example [13]. In the case of this study, for the expected healthiness of the samples, the difference between the evaluations of the labels did not reach statistical significance (*p* > 0.05) (Figure 4A). A probable explanation for this fact may be due to yogurt, in general, is commonly considered a healthy product by consumers, regardless of the sweetening agent or the labeling of this product. Huang and Lu [40], in products classified as utilitarian (cereals, yogurt, and milk), found that products labeled blue were perceived as healthier than when labeled red, as well as Schuldt [41], in chocolates labeled with green color, differing from the results found in this study.

Regarding the expected acceptance, the R1 label (orange color sweetened with stevia/thaumatin sweeteners) did not differ statistically (*p* > 0.05) from the R6 label (orange color sweetened with sucrose). However, the expected acceptance of the R1 label was significantly higher (*p* < 0.05) when compared to four of the five labels that contained sucrose (white, yellow, blue and green) (M = 6.78 versus 6.51, 6.39, 6.43, 6.43 and 6.35 respectively; Figure 4B). Information about a reduction in ingredient content related to expected sensory characteristics influences hedonic expectations of consumers [42]. In the case of this study, the information “without added sugar” presented on labels with stevia/thaumatin sweeteners influenced the expectation of consumers, consistent with previous studies that state that products that contain a claim of “no added sugar” are generally preferable, as it refers to something more natural [43]. With increasing consumer interest in the negative effects of excess sugar on the human diet, information on sugar content (reduced or without) can have a positive effect on consumer expectations.

Color of label also appears to have influenced consumer expectations. Because the color of the yogurt mentioned on the label refers to orange (which is the color of mango fruit), consumers possibly preferred the label more associated with the color that the product would have. This is due to the fact that consumer expectations are generated through previous experiences with the product, information presented on the label, characteristics of the packaging, and the product itself through its appearance [44]. Lick et al. [11], in studies with wine labels, suggested that in order to make consumers expect a fruity and sweet taste, orange color seems to be more effective. Characteristics of packaging must create sensory and hedonic expectations that reflect the real characteristics of the food to avoid any dissatisfaction when consumers go to consume the product [11,44]. Ares and Deliza [15] also found that color had an influence on consumer expected liking and willingness to buy for dairy dessert packaging, with consumers preferring packaging with colors associated with sweet milk desserts such as vanilla and *dulce de leche*.

### 3.4. Cluster Analysis and Preference Map

**Figure 5:**
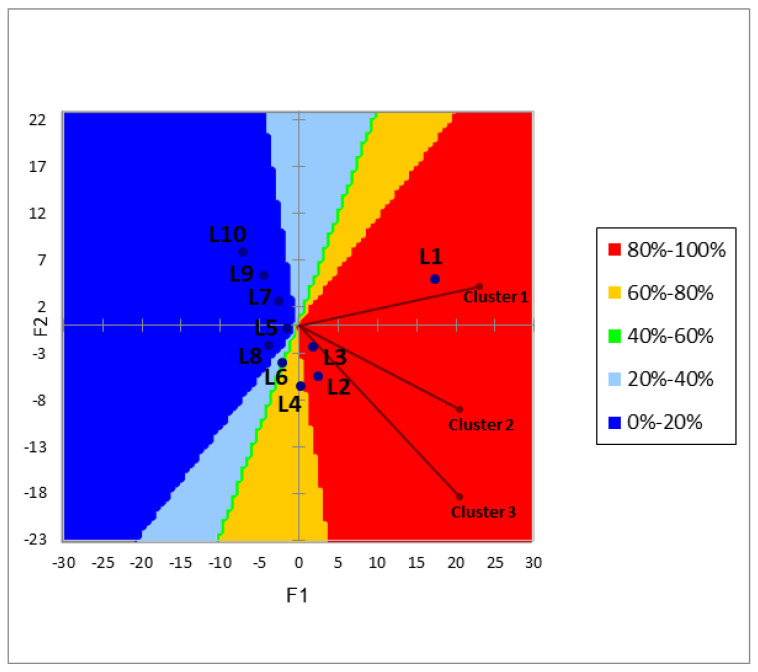
Preference map of consumers clusters and contour plot of preference for yogurt labels (Blue points): L1 (Orange color label with sweeteners), L2 (White color label with sweeteners), L3 (Yellow color label with sweeteners), L4 (Blue color label with sweeteners), L5 (Green color label with sweeteners), L6 (Orange color label with sucrose), L7 (White color label with sucrose), L8 (Yellow color label with sucrose), L9 (Blue color label with sucrose) and L10 (Green color label with sucrose).

In order to verify whether the 151 consumers participating in the study formed preference groups, HCA was applied. Consumer segmentation based on acceptance data provided three groups of individuals: cluster one (59.6% of evaluators), cluster two (30.5% of evaluators), and cluster three (9.9% of evaluators). The results of the preference mapping performed on the general acceptance data are shown in Figure 5. Contour graphs show the preferential zones in relation to the labels, where red zones are the ones with the highest preference region (80 to 100%), followed by the yellow region (60 to 80%), the green region (40 to 60%), and the light blue region (20 to 40%) with the dark blue region (0 to 20%) being the lowest preferred region.

Clusters (1, 2 and 3) preferred labels sweetened with stevia/thaumatin sweeteners. L1, L2 and L3 labels had a greater range of preference, reaching 80 to 100% of consumers. These labels had the information that they were sweetened with stevia/thaumatin sweeteners. Only the green color label showing it was sweetened with stevia/thaumatin sweeteners (L5) was in the least preferred zone (0 to 20% of consumers). The information that the label was sweetened with sucrose led to less consumer preference, with these labels being in the less preferred regions (L6 20% to 40% of consumers; L7, L8, L9 and L10 0% to 20% of consumers), corroborating the results of the ANOVA and Tukey’s test (Figure 4A,B).

In this study, consumer preference expectations for skyr yogurt labels containing sweeteners were higher than for those with sucrose. However, sensory tests with skyr yogurt formulations sweetened with stevia, thaumatin, and sucrose revealed that formulations sweetened with the natural sweetener stevia were less preferred compared to yogurts sweetened with sucrose [9,34].

## 4. Conclusions

The results of this study showed that extrinsic cues related to yogurt labels affected consumer acceptance expectations. The information regarding sweetener type contained in the labels was a factor of difference among the samples. Labels containing the information “without added sugar” had higher percentages of expectation of ideal sweetness and consumer acceptance. Colors had an impact on acceptance but not on the expectation of healthiness. In general, buying intention followed the same trend as ideal of sweetness, with lower percentage of willingness to buy for sucrose labels.

The study also showed important determinants of the choice of concentrated yogurt for the consumer at the time of purchase. Skyr yogurt is still unknown by the population but shows itself as an alternative to concentrated yoghurt with a healthy appeal.

The study was limited by the fact that consumers did not sensorially assess the samples. However, this issue was considered previously, to specifically verify the consumer’s expectation before the visual, aroma, flavor, and texture experience of the product. The results obtained can help guide food products, such as the study carried out with skyr yogurt.

## Figures and Tables

**Table 1:** Demographic information of survey participants.

Characteristic	Frequency	%
Gender		
Female	108	71.5
Male	40	26.5
Non-binare	0	0
Rather not answer	2	1.3
Other	1	0.7
Age (years)		
18–24 old	13	8.6
25–34 old	68	45.0
35–44 old	51	33.8
45–59 old	14	9.3
60 and above	5	3.3
Marital status		
Single	90	59.6
Married	53	35.1
Divorced	8	5.3
Widower	0	0
Educacion		
Elementary school	1	0.7
High school	6	4.0
Undergraduate	16	10.6
Graduate	16	10.6
Postgraduate	111	73.5
Rather not answer	1	0.7
Income (number of minimal Brazilian wages)		
Up to 1	11	7.3
1 to 2	18	11.9
>2 to 5	42	27.8
>5 to 10	47	31.1
>10	24	15.9
Rather not answer	9	6.0
Brazilian region		
North	4	2.6
Northeast	66	43.7
Midwest	10	6.6
Southeast	63	41.7
South	8	5.3
Yogurt consumption		
Never	3	2.0
Occasionally	56	37.1
Monthly (at least once per month)	34	22.5
Weekly (at least once per week)	37	24.5
Daily	21	13.9
Concentrated yogurt consumption		
Never	13	16.6
Occasionally	56	58.3
Monthly (at least once per month)	10	11.9
Weekly (at least once per week)	12	10.6
Daily	1	2.6

## Data Availability

Data is contained within the article.
